# The Role of Microglia in Bacterial Meningitis: Inflammatory Response, Experimental Models and New Neuroprotective Therapeutic Strategies

**DOI:** 10.3389/fmicb.2019.00576

**Published:** 2019-03-25

**Authors:** Sigrun Thorsdottir, Birgitta Henriques-Normark, Federico Iovino

**Affiliations:** ^1^Department of Microbiology, Tumor and Cell Biology, Karolinska Institutet, Bioclinicum, Stockholm, Sweden; ^2^Department of Clinical Microbiology, Karolinska University Hospital, Stockholm, Sweden; ^3^Singapore Centre for Environmental Life Sciences Engineering (SCELSE) and Lee Kong Chian School of Medicine (LKC), Nanyang Technological University (NTU), Singapore, Singapore

**Keywords:** microglia, bacterial meningitis, blood-brain barrier, experimental models, therapeutic strategies

## Abstract

Microglia have a pivotal role in the pathophysiology of bacterial meningitis. The goal of this review is to provide an overview on how microglia respond to bacterial pathogens targeting the brain, how the interplay between microglia and bacteria can be studied experimentally, and possible ways to use gained knowledge to identify novel preventive and therapeutic strategies. We discuss the dual role of microglia in disease development, the beneficial functions crucial for bacterial clearing, and the destructive properties through triggering neuroinflammation, characterized by cytokine and chemokine release which leads to leukocyte trafficking through the brain vascular endothelium and breakdown of the blood-brain barrier integrity. Due to intrinsic complexity of microglia and up until recently lack of specific markers, the study of microglial response to bacterial pathogens is challenging. New experimental models and techniques open up possibilities to accelerate progress in the field. We review existing models and discuss possibilities and limitations. Finally, we summarize recent findings where bacterial virulence factors are identified to be important for the microglial response, and how manipulation of evoked responses could be used for therapeutic or preventive purposes. Among promising approaches are: modulations of microglia phenotype switching toward anti-inflammatory and phagocytic functions, the use of non-bacterolytic antimicrobials, preventing release of bacterial components into the neural milieu and consequential amplification of immune activation, and protection of the blood-brain barrier integrity.

## Microglia in Bacterial Meningitis: Destructive or Helpful?

Bacterial meningitis is a life-threatening disease, predominantly caused by *Streptococcus pneumoniae*, *Neisseria meningitidis*, and *Haemophilus influenzae* type b (Hib) in children and adults, and *Escherichia coli* K1 in neonates. Blood-borne bacteria invade and infect the brain tissue, resulting in a severe inflammatory response in the brain parenchyma and meninges (Iovino et al., 2016b). Current preventive and therapeutic strategies, including antibiotics and vaccines, have substantially improved the clinical outcome of bacterial meningitis, but the disease still represents a significant threat. This is highlighted by the emergence of resistant bacterial strains and rise of non-vaccine type strains (Doran et al., 2016). The disease burden varies between geographical regions, and prognosis is dependent on availability of health care and efficiency of vaccination programs (Grandgirard and Leib, 2010). Survivors of bacterial meningitis often suffer from neurological damage such as hearing loss, motor and cognitive impairment, largely caused by the host inflammatory response itself. While the resulting physical impairment often improves over time, the cognitive decline can persist lifelong especially in pediatric cases due to the sensitivity of the developing brain (Hoogman et al., 2007; Grandgirard and Leib, 2010).

Several mechanisms involving intrinsic virulence factors of bacteria and host defenses facilitate brain invasion and tissue injury (Gerber and Nau, 2010; Doran et al., 2016; Iovino et al., 2016a, 2017). Microglial cells are the most abundant and well-studied myeloid population of the Central Nervous System (CNS). They are ontogenetically distinct from other hematopoietic stem cell derived brain macrophages, as they arise in an earlier embryonic stage from yolk sac progenitors (Prinz and Priller, 2014). Microglias reside in the brain parenchyma and are considered the immune sentinels of the brain (Greter et al., 2015; Michels et al., 2015; Barichello et al., 2016). Once the bacteria gain access to the brain after breaching the blood-brain barrier (BBB), bacterial products such as lipopolysaccharides from Gram-negative bacteria and lipoteichoic acid from Gram-positive bacteria, are recognized by pattern recognition receptors (PRR) on microglia (Doran et al., 2016; Iovino et al., 2016a). Examples of other receptors expressed by microglia, involved in evoking inflammatory responses via damage signals derived from injured host cells, are purinergic receptors, tachykinin receptors, estrogen receptors (ER) and cannabinoid receptors (CB) (Stella, 2010; Burmeister et al., 2017; Tohidpour et al., 2017; Lee et al., 2018). Microglia can also serve as antigen presenting cells by bacterial processing and MHC class II upregulation, and together with astrocytes, microglia represent the primary source of complement in the brain (Almolda et al., 2011; Veerhuis et al., 2011).

Upon activation microglia undergo morphological changes, draw in their long ramifications associated with surveilling functions and obtain a larger amoeboid shape (Kettenmann et al., 2011; Iovino et al., 2013). In a reactive state, microglia release an array of soluble factors and exhibit migratory, proliferative and phagocytic properties adjusted to the type of activating stimuli. Reactive microglia can be further divided into M1 and M2 polarized states; the M1 “classically activated” phenotype is characterized by release of pro-inflammatory factors and the M2 “alternatively activated” is associated with enhanced phagocytosis and release of anti-inflammatory and neurotrophic factors (Cherry et al., 2014; Orihuela et al., 2016). However, such dichotomous classification is now considered oversimplified as microglia exists on a spectrum of functional phenotypes (Sousa et al., 2017). A more accurate framework to classify microglial phenotypic states under healthy, diseased and developmental stages remains to be agreed upon (Ransohoff, 2016; Holtman et al., 2017). The release of pro-inflammatory cytokines such as tumor necrosis factor (TNF)-α and interleukin (IL)-6, and chemokines leads to increased permeability of the brain microvascular endothelium and upregulation of adhesion molecules, facilitating leukocyte recruitment into the brain (Gerber and Nau, 2010; Mook-Kanamori et al., 2011; Coutinho et al., 2013; Tohidpour et al., 2017). Activated microglia further contributes to leakage and breakdown of the BBB by producing reactive oxygen and nitrogen species, and matrix metalloproteases (MMP) (da Fonseca et al., 2014). The increased intracranial pressure and the self-amplifying inflammatory environment ultimately results in respiratory burst and neuronal cell death (Coutinho et al., 2013; Doran et al., 2016).

Microglial activation, in particular the M1 skewed response, is a hallmark of neuroinflammation and thus microglia are fundamental players in the pathophysiology of bacterial meningitis. In the latest years, an extensive effort has been put into scrutinizing molecular mechanisms of microglial responses to brain pathogens. A handful of therapeutic strategies have been discovered and evaluated in experimental settings (Barichello et al., 2016). Attractive targets to manipulate the microglial response include modulation of phenotype switching toward M2, enhancing phagocytic functions, blocking pro-inflammatory mediators, promoting release of neurotrophic factors and protecting the BBB integrity (Hu et al., 2015). Here we discuss recent advances made in our understanding of how microglia react to bacteria and vice versa, and potential implication for improved therapeutic strategies.

## Models and Considerations When Studying Microglial Reactivity to Pathogenic Bacteria

Patient derived post-mortem brain biopsies have provided valuable insights into the pathophysiology of bacterial meningitis, but these are only representative for fatal disease (Iovino et al., 2017). In the past few years several *in vivo* models have been developed, including mice, rat and rabbit models, inoculated with bacteria either intranasally, intravenously, intrathecally or by other routes (Koedel and Pfister, 1999; Hoogland et al., 2015; Iovino et al., 2016b). Disease development is usually monitored by clinical scoring of symptoms and cerebrospinal fluid (CSF) diagnosis. Pathogen inoculation into the cisterna magna and CSF sampling are more readily performed on rabbits and rats than mice, due to their larger size (Paul et al., 2003). However, mouse models represent other advantages reflected by availability of genetically modified models and biological reagents to study relevant pathways.

Several *in vitro* models of microglia exist to study microglia characteristics. Those include the immortalized cell lines BV2 (murine), HAPI (rat) and HMC3 (human) (Dello Russo et al., 2018; Timmerman et al., 2018). Primary cultures of freshly isolated microglia and stem cell-derived microglia represent a better biological model than cell lines in mimicking normal physiology of microglia (Timmerman et al., 2018). *Ex vivo* microglial response has been studied using organotypic slice cultures from mice, hamsters, rats and non-human primates (Czapiga and Colton, 1999; Burmeister et al., 2017). Microglia display a broad spectrum of different phenotypes, they are involved in different functions, and are sensitive to changes in the neural environment and adjust accordingly. Hence, the transcriptomic signature changes dramatically after a few days of culturing outside of their normal physiological niche which represents a challenge, especially when studying or comparing to homeostatic state of microglia (Galatro et al., 2017). Moreover, established protocols for microglial isolation and cell sorting have been reported to lead to artifacts in downstream analysis, by contamination of ingested material from the surroundings and induction of activation signature. RiboTag translatome profiling, a recently developed technique for gene expression analysis, has been suggested to limit artifacts and improve accuracy when studying functional states of microglia (Haimon et al., 2018).

The majority of the studies on microglial responses to peripheral inflammatory stimuli, or direct bacterial stimuli, have been conducted in mice, whereas activation has been determined by measuring ionized calcium-binding adapter molecule 1 (Iba-1), CD68, CD11b or Toll-like receptor (TLR) surface expression and TNFα and IL1β mRNA protein levels (Hoogland et al., 2015). In addition, the use of knockout mice in experimental meningitis, deficient in key cytokines, adhesion molecules and proteases, has contributed to a more complete understanding of the involvement of inflammatory pathways and which steps may be targeted for therapeutic purposes (Paul et al., 2003).

Until recently it has been difficult to distinguish microglia from other related myeloid cells due to large overlaps in genetic signature and surface markers. TMEM119 is the first marker discovered which is exclusively expressed by microglia in healthy and diseased states, in both mice and humans, opening up new venues for microglia research (Bennett et al., 2016). Other potential microglia-specific markers discovered by recent transcriptomic profiling, include P2Y12, Siglec-H, olfactomedin-like 3 and Sal1 (Colonna and Butovsky, 2017; Holtman et al., 2017). Transgenic mice with GFP labeled microglia, under the promoter of the fractalkine receptor or Iba-1, have been generated and are excellent tools for imaging the healthy brain, but less useful when studying disease states since both the fractalkine receptor and Iba-1 are expressed by peripherally derived cells present in the brain under pathological conditions (Jung et al., 2000; Hirasawa et al., 2005). Likewise, depletion of microglia has been challenging. After partial or complete eradication of microglia, other cells such as peripherally derived monocytes serve as microglia progenitors and repopulate the brain shortly after depletion (Lund et al., 2018). Sustained microglia depletion can be achieved by continuous pharmacological administration of inhibitors of colony-stimulating factor 1 receptor (CSF-1R). Microglia are the only immune cells in the CNS that express CSF-1R, and microglial development and survival is highly dependent on CSF-1R signaling (Chitu et al., 2016; Han et al., 2017). In contrast, liposomal clodronate microinjections into selected anatomical regions of the brain have been used to selectively deplete microglia within that region (Torres et al., 2016). Very recently, the specific inhibitor of colony-stimulating factor 1, PLX5622, was administered dietary to continuously deplete microglia in two studies of viral encephalitis. In both studies microglia were found to have a neuroprotective role (Waltl et al., 2018; Wheeler et al., 2018) Finally, zebrafish is emerging as a model for studying the functionality and development of microglia (Lyons and Talbot, 2014).

Appropriate modeling and availability of relevant markers is crucial to advance our understanding of the microglia-pathogen interplay. Animal models have proven useful to study the pathogenesis of bacterial meningitis, and identify and evaluate potential novel therapeutic targets. The inherent complexity of microglia, reflected by the heterogeneity of activation states, demands careful consideration of choice/combination of markers and models when monitoring physiological and pathophysiological responses of this multi-tasking cell type.

## Exploring Therapeutic Targets: Inflammatory Signaling, Phagocytosis, Protecting the Blood-Brain Barrier Integrity

**FIGURE 1:**
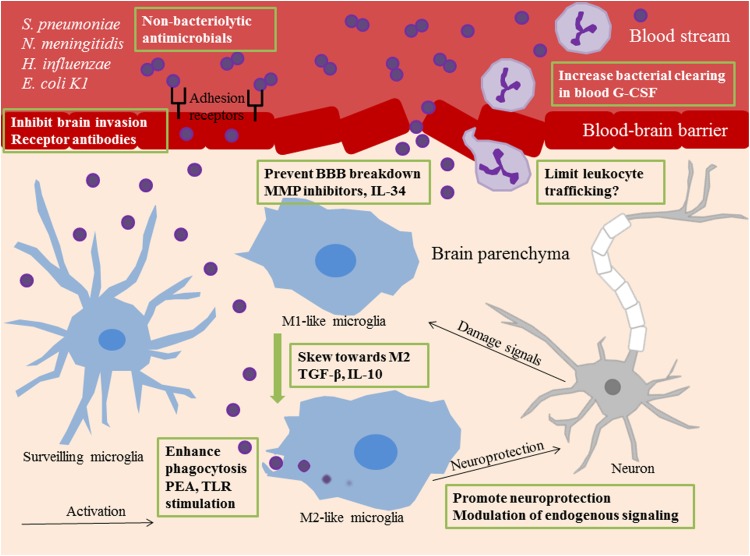
Summary of novel therapeutic targets in bacterial meningitis. Different therapeutic strategies are shown within green frames. Surveilling microglia recognize invading bacteria or bacterial components and undergo activation. M1 polarized microglia are potent propagators of neuroinflammation. M2 polarized microglia have phagocytic and anti-inflammatory properties. In bacterial meningitis, phenotypical shift toward M2 functions can be acquired by administration of the anti-inflammatory cytokines TGF-β and IL-10. Phagocytic uptake of bacteria by microglia can be enhanced with prophylactic TLR stimulation or PEA pretreatment. The use of non-bacteriolytic antimicrobials prevents release of bacterial components into the external milieu which can feed the inflammatory response. Blockade of receptors on the blood brain endothelium prevents bacterial invasion of the brain. BBB breakdown is a consequence of neuroinflammation and can be limited with MMP inhibitors and IL-34 administration. G-CSF increases neutrophil numbers in the circulation and enhances bacterial clearing from the blood. Pharmacological blockade of leukocyte trafficking into the brain can be deleterious for disease outcome, but different approaches in limiting infiltration may be more successful. Neurogenesis and neuroprotection can be promoted via modulation of endogenous signaling pathways. TGF-β, Transforming growth factor beta; IL-10, interleukin-10; TLR, Toll-like receptor; PEA, Palmitoylethanomide; BBB, blood-brain barrier; MMP, matrix metalloproteases; IL-34, interleukin-34; G-CSF, granulocyte colony-stimulating factor.

Various anti-inflammatory, blocking agents, and prophylactic stimulators targeting relevant signaling events, summarized in Figure 1, have shown promising results in promoting survival and preventing injury in experimental bacterial meningitis.

Inhibition of deleterious functions of microglia, associated with the classically activated phenotype M1, represents an attractive option for adjuvant therapy to antimicrobials (Barichello et al., 2015, 2016). Enhancement of the neuroprotective functions of homeostatic and the alternatively activated phenotype M2 microglia has been explored as therapeutic option in many neurological diseases, including bacterial meningitis (Cherry et al., 2014; Barichello et al., 2016; Chen and Trapp, 2016). Several strategies for skewing the inflammatory responses toward anti-inflammatory functions have been applied in experimental settings, in rodents and cell cultures *in vitro*, and also in the clinic in the case of corticosteroid and glycerol (Gerber and Nau, 2010). Efficacy of adjuvant Corticosteroid treatment has been debated, but has only been proven beneficial in adult patients with pneumococcal meningitis in high-income countries (Liechti et al., 2015). The anti-inflammatory cytokine IL-10 was shown to inhibit production of inflammatory cytokines in microglia and astrocytes isolated from mice previously challenged with the Gram-negative bacteria *Borrelia burgdorferi* and *N. meningitidis*, injected into the cerebroventricular space (Rasley et al., 2006). Induction of microglia of the M2 phenotype is dependent on TGF-β signaling, and it has been proposed that activated astrocytes act as source of TGF-β in the presence of IL-10, which in turn attenuates pro-inflammatory cytokine production by microglia and upregulation of anti-inflammatory mediators (Zhou et al., 2012; Norden et al., 2014). Recently, therapeutic effects of TGF-β administration were evaluated in the context of hemorrhagic stroke. TGF-β was injected directly into the brains of mice with induced intracerebral hemorrhage, and microglial responses were recorded. Microglia showed an overall dampened inflammatory profile and the treated mice underwent a quicker functional recovery. The same effects were observed in stroke patients, whereas initial increased plasma TGF-β concentrations were an indicator for improved clinical outcome (Taylor et al., 2017). TGF-β could therefore be a strong candidate as a future therapeutic agent in the treatment of acute brain injury, including bacterial meningitis. However, since activation of TGF-β has been postulated to have a role in hydrocephalus development, through induction of subarachnoid fibrosis, this approach may hold some drawbacks (Botfield et al., 2013). Microglia express PRRs able to recognize bacterial products, these include TLRs and NOD-like receptors (NLRs). NOD2, a member of the NLR family, has been shown to be crucial in propagating inflammation in response to Gram-positive and Gram-negative bacteria (Liu et al., 2010). TLR1/2, TLR4 and TLR9 have been studied most extensively in experimental bacterial meningitis. Prophylactic stimulation of these receptors resulted in increased phagocytic uptake and intracellular killing of *S. pneumoniae* and *E. coli* K1 by microglia (Ribes et al., 2009, 2010). Inhibition of TLR signaling has also been explored. The small molecule drug ibrutinib, a tyrosine kinase inhibitor, was shown to regulate TLR4 signaling in microglia by dampening LPS induced pro-inflammatory responses (Nam et al., 2018). The protein tyrosine kinase inhibitor AG126 reduced disease severity induced by pneumococcal cell walls in a similar way by blocking leukocyte influx to the brain and TNFα production (Hanisch et al., 2001). One promising novel therapeutic approach is TLR stimulation in combination with a TGF-β receptor agonist. When treated with these compounds, primary microglia cells have shown increased phagocytic capacity and clearing of *E. coli* K1 without increasing release of proinflammatory cytokines or affecting cell viability (Diesselberg et al., 2018).

The role of the endogenous receptor for the tachykinin substance P, the neurokinin 1 (NK_1_) receptor, has recently been described as a potent propagator of neuroinflammation and neurotoxicity. NK_1_ receptor antagonists have been used as antidepressant and antiemetic drugs (Brain and Cox, 2006). Upon challenge with *S. pneumoniae* in mice, administration of the receptor antagonists led to reduced inflammation and increased protection of the brain (Burmeister et al., 2017). Distinctively, activation of ER-β is associated with neuroprotection through induction of brain-derived neurotrophic factor (BDNF). Some ginsenosides, bioactive molecules derived from the ginseng plant, can work as ER-β agonists. Ginsenoside Rb1 treatment of mice with experimental meningitis rescued tissue damage to some extent, with greater effect in female mice (Lee et al., 2018). The protective properties of signaling through ER might explain the gender bias in cognitive impairment persistency after bacterial meningitis, where the male sex is a risk factor (Hoogman et al., 2007).

Stimulation of phagocytic functions of microglia could help clearing the brain from bacteria quicker and in that way resolve inflammation. Palmitoylethanomide (PEA) is an endogenous lipid that has been shown to have anti-inflammatory effects on various immune cells. In experimental meningoencephalitis or sepsis induced by *E. coli* K1 in mice, pretreatment with PEA resulted in improved survival. Microglia and macrophages pre-stimulated with PEA *in vitro* showed a dose-dependent higher uptake and intracellular killing of *E. coli* K1 bacteria (Redlich et al., 2014). PEA was recently shown to mediate its effect on microglial activation via the endocannabinoid system, promoting CB2 expression on microglia (Guida et al., 2017).

Several experimental therapeutic interventions aim at protecting the integrity of the BBB and limit leukocyte invasion into the brain, and thus prevent excessive microglial responses (Liechti et al., 2015). These include pharmacological inhibition of MMPs, in particular MMP9, which is elevated in the CSF of meningitis patients and implicated in BBB breakdown and associated with neurological sequela in bacterial meningitis. MMP inhibition, sometimes in combination with TNFα inhibitors, have neuroprotective effects in rat models of experimental bacterial meningitis (Grandgirard and Leib, 2010). The cytokine IL-34, mainly expressed by damaged neurons in the CNS, can bind to the CSF-1R receptor of microglia and was recently found to upregulate tight junctions in brain endothelial cells as well (Jin et al., 2014). We recently proposed that blockade of the BBB endothelial receptors PECAM-1 and pIgR, which the pneumococcus uses to invade the brain, could be a novel therapeutic approach. Mice treated with blocking antibodies in combination with ceftriaxone antibiotic reached a survival rate of 100% in a 10 day survival study, which was also characterized by attenuation of microglial activation (Iovino et al., 2018). Leukocyte trafficking into the brain contributes to destruction of the BBB, and recruited neutrophils contribute to elevated MMP levels. Blocking chemotaxis of immune cells into the brain was therefore suggested to have applications in preventing brain injury. However, survival of rats with experimental pneumococcal meningitis treated with fucoidin, an inhibitor of leukocyte rolling, was compromised reflected by elevated bacterial load in the blood and inability to clear the infection (Brandt et al., 2005). Recently, adjuvant granulocyte colony-stimulating factor (G-CSF) therapy, which increases the numbers of neutrophils in the circulation, was shown to have positive effects on spatial learning and neurogenesis in mice with experimental pneumococcal meningitis. G-CSF treatment had no effect on microglia or astrocyte activation (Schmidt et al., 2015).

In the case of the Gram-positive *S. pneumoniae* the polysaccharide capsule is one of the most potent activator of microglial pro-inflammatory responses via PPR signaling. In fact, administration of cell walls only was enough to generate CNS inflammation in mice (Tuomanen et al., 1985). The capsule confers protection against killing by the immune system, and in microglia encapsulated bacteria are phagocytized, but resistant to intracellular killing. Intracellular pneumococci were found to upregulate capsular genes and were not fused with phagolysosomes (Peppoloni et al., 2010, 2013). The pneumococcal surface protein PspA and the novel Spr1875 were shown to be involved in protecting bacteria from intracellular killing using mutant strains (Peppoloni et al., 2013; Ricci et al., 2013). Another major pneumococcal virulence factors is pneumolysin which has been associated with neuronal and microglial cell death (Braun et al., 2002). *S. pneumoniae* was found to induce pyroptosis in microglial cells in a pneumolysin dependent manner, partially counteracted by increased expression of autophagy related genes (Kim et al., 2015). A current standard treatment of pneumococcal meningitis is ceftriaxone, a cephalosporin antibiotic belonging to the β-lactam family. β-lactam antibiotics induce lysis of bacteria, leading to release of bacterial components such as pneumolysin and pneumococcal cell wall, which in turn further augments microglial activation and neuroinflammation. The use of a potent bactericidal, but non-bacteriolytic antibiotic, could therefore protect the brain from further immune activation and prevent neurological damage (Koedel et al., 2010; Liechti et al., 2015). An example of such an agent is daptomycin, which has been evaluated in a rabbit and infant rat experimental model of pneumococcal meningitis and found to have favorable characteristics in bacterial killing efficiency and attenuation of neuroinflammation (Stucki et al., 2007; Grandgirard et al., 2010). However, since daptomycin cannot cross the outer membrane of Gram-negative bacteria its bactericidal activity is restricted to Gram-positive bacteria (Randall et al., 2013). Another antibiotic which may have an application as adjuvant meningitis therapy is the tetracycline antibiotic minocycline, which was shown to have anti-inflammatory and neuroprotective properties in rats challenged with LPS, proposedly via inhibition of microglial activation (Zhu et al., 2014). Two recent studies explore the therapeutic advantage of using non-bacteriolytic antibiotic in combination with a MMP inhibitor or as adjunctive ceftriaxone treatment, in combination with anti-complement C5. Both studies reported improved neurofunctional outcome in mice and rats after experimental pneumococcal meningitis (Muri et al., 2018; Klein et al., 2019). Many factors affect the selection of antimicrobial therapy in bacterial meningitis, type of pathogen and respective bacteriostatic/bactericidal efficiency, BBB penetration and antibiotic resistance perspectives. The recent notion of how the release of bacterial components into the neural environment, which activates microglia to potentiate neuroinflammation, is likely to affect future therapeutic regimens for bacterial meningitis. Hence, this important aspect of the pathophysiology of bacterial meningitis should be considered when screening for novel treatment approaches or when revisiting older ones like minocycline (Whitby and Finch, 1986).

## Concluding Remarks

Taken together, a growing body of evidence implicates microglia as central players in the pathogenesis and resolution of bacterial meningitis. High mortality and morbidity rates and imminent emergence of antimicrobial resistant cases highlight the need to identify and evaluate novel therapeutic targets. Employment of improved disease models and new tools to study the dynamics of microglial responses across diverse pathological conditions, represent great advantages for future studies. Our increasing knowledge of the pathophysiology of bacterial meningitis, and bacterial and host factors involved, will certainly contribute to the goal of improved preventative and therapeutic approaches in the management of bacterial meningitis.

## Author Contributions

ST and FI wrote the literature study and wrote the manuscript. BH-N contributed in writing the manuscript.

## Conflict of Interest Statement

The authors declare that the research was conducted in the absence of any commercial or financial relationships that could be construed as a potential conflict of interest.
